# Modeling and Epitaxial Growth of Homogeneous *Long*-InGaN Nanowire Structures

**DOI:** 10.3390/nano11010009

**Published:** 2020-12-23

**Authors:** Sung-Un Kim, Yong-Ho Ra

**Affiliations:** 1 Optic & Electronic Component Material Center, Korea Institute of Ceramic Engineering & Technology, Jinju 52851, Korea; sungunkim26@gmail.com; 2 Division of Materials Science and Engineering, Hanyang University, 222, Wangsimni-ro, Seongdong-gu, Seoul 04763, Korea

**Keywords:** nanowire, InGaN, molecular beam epitaxy, green gap, LED

## Abstract

One-dimensional nanowires based on Group III-nitride materials are emerging as one of the most promising structures for applications of light-emitting diodes (LEDs), laser diodes (LDs), solar cells, and photocatalysts. However, leading to the so-called “green gap” in photonics, the fabrication of high concentration indium gallium nitride (InGaN) and *long*-InGaN structures remains still challenging. In this study, we performed simulations for structural modeling of uniform temperature distribution in a nanowire epitaxy, and have successfully developed high-concentration InGaN and *long*-InGaN nanowire heterostructures on silicon (Si) substrate using molecular beam epitaxy (MBE) system. From scanning electron microscope (SEM) and transmission electron microscope (TEM) results, it was confirmed that the various doped-InGaN nanowire structures show much higher crystal quality compared to conventional nanowire structures. By introducing a new three-step modulated growth technique, the *n*-/*p*-InGaN active regions were greatly increased and the optical properties were also dramatically improved due to reduced phase separation. In addition, a multi-band *p*-InGaN/GaN heterostructure was successfully fabricated with the core–shell nanowire structures, which enable the emission of light in the entire visible spectral range, and protect the InGaN surface from surface recombination. This paper offers important insight into the design and epitaxial growth of InGaN nanowire heterostructures.

## 1. Introduction

One-dimensional (1D) semiconductor nanowire structure, because of excellent structural, optical, and electrical properties, is recognized as a core technology that can overcome the limitations of existing technologies in optoelectronics, display, bio, and environment fields [1,2,3]. However, in order to fabricate nano-devices and systems based on 1D semiconductor nanowires, there are still various difficulties to be solved [4]. In particular, synthesis technology that can accurately control chemical composition, structure, and size, and optimal nano-fabrication technology that can precisely control various nano-quantum structures are essential for the application of next-generation nano-based technology. Group III-nitride based nanowire semiconductors not only are direct transition materials but also can adjust the bandgap energy from 0.7 eV to 6.2 eV, so it is promising for LEDs, LDs, solar cells, and photocatalysts applications [5]. Moreover, the nitride-based nanowire structure has a nearly dislocation-free structure due to effective lateral stress relaxation, and has the advantages of reduced polarization fields, large surface area, and tunable emission wavelength [6,7,8,9]. Ultimately, such nanowire structures can lead to enhancing internal and external quantum efficiency, and reducing defect-assisted Auger recombination [9,10,11]. However, in the conventional nitride-based nanowire structures, surface recombination occurs due to non-uniform nanowire morphology, and severe inhomogeneous distribution is caused in InGaN structure as increases the concentration of indium (In) [12,13,14]. This non-uniform distribution of the In concentration causes enormous obstacles to the performance of optical and electrical devices [15].

In general, as the In concentration in InGaN structures increases, a “green gap” occurs in which the quantum efficiency is significantly decreased due to the poor InGaN material quality. Consequently, this leads to limitations in fabricating high-efficiency green or red photonic devices [16,17]. Furthermore, it is necessary to maximize the absorption efficiency by increasing the active region in photocatalyst applications for water splitting and solar cell, but it is still very difficult to increase the In concentration in InGaN and to fabricate a large-area InGaN structure [18,19]. So far, a critical issue remains that an epitaxial growth technique for fabricating *long*-InGaN nanowire structures has not been developed yet. In this context, we introduced a new nanowire epitaxial growth technique that can fabricate *long*-InGaN heterostructures, and have also shown structural modeling in nanowire systems using a heat transfer simulation.

In this study, we investigated various crystal growth and optical/structural properties of high-aspect-ratio GaN-based nanowires operating in the entire visible spectral range. The nanowire structures were fabricated on a Si (111) substrate using a catalyst-free deposition technique by a molecular beam epitaxy (MBE) system. It was confirmed that high density and high-aspect-ratio GaN nanowires were grown vertically on the Si substrate along the <0001> direction. By introducing a new 3-step temperature modulation growth technique, the InGaN active region was remarkably increased, and *n*-/*p*-type doped *n*-InGaN and *p*-InGaN structures were successfully fabricated. In addition, it was confirmed that such a growth technique can significantly reduce the phase separation and can dramatically improve the optical properties. From SEM and TEM results, it is shown that the developed nanowire heterostructures have high-quality crystalline compared to conventional nanowire structures. Additionally, a high-quality multi-band nanowire, which can emit the entire visible spectral range, was successfully grown with a core–shell structure that can protect the InGaN surface from surface recombination. Such InGaN nanowire heterostructures will be very useful for the applications of LEDs, LDs, solar cells, and photocatalysts.

## 2. Experimental Details, Results and Discussion

**Figure 1:**
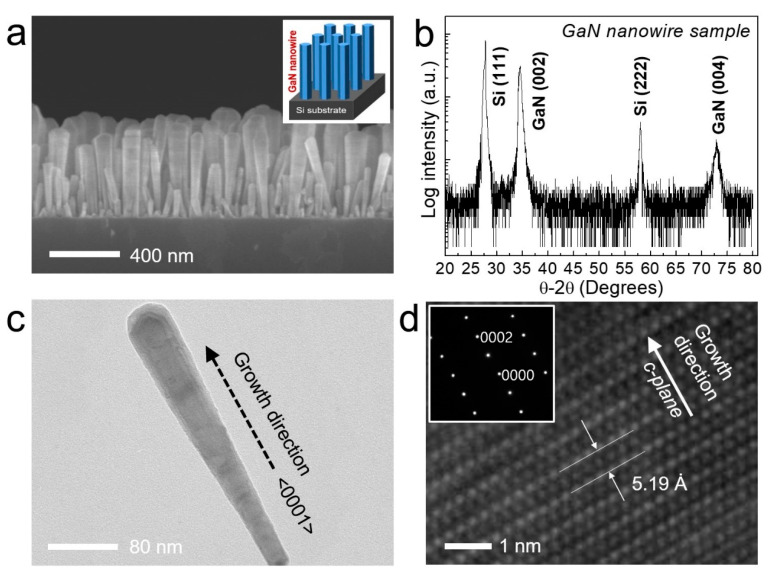
(**a**) A cross-sectional field-emission SEM (FE-SEM) image of undoped gallium nitride (GaN) nanowires grown on a Si (111) substrate by the catalyst-free method. (**b**) X-ray Diffraction (XRD) pattern of GaN nanowires grown on the Si substrate. (**c**) A bright-field low-magnification TEM image of a single GaN nanowire. (**d**) HR-TEM lattice image and selected area diffraction (SAED) patterns of defect-free single-crystalline GaN structure.

We first designed GaN nanowire structures on the substrate, shown in the inset of Figure 1a. Here, a Si (111) substrate and a plasma-assisted MBE system are used for the fabrication of high-quality nanowires. In the first step, the gallium (Ga) source was supplied for 15 s at a temperature of 680 °C to form the seeding layer of GaN nanowires. The Ga seed nano-droplets can help to nucleate GaN nanowires on the substrate. GaN nanowires were grown at the substrate temperature of 710 °C with Ga beam equivalent pressure (BEP): ~5.3 × 10^−8^ Torr in a nitrogen (N_2_) atmosphere of 0.5 standard cubic centimeter per minute (sccm). The plasma power during the epitaxial growth was fixed at 350 W.

A tilted-view field-emission SEM (FE-SEM) image of the undoped GaN nanowires grown on a Si substrate by MBE system is shown in Figure 1a. Undoped GaN nanowires were formed with high density and uniform thickness in a nearly vertical direction. The average diameter of the nanowires is about 40–60 nm, and the length is about 400–600 nm. As shown in Figure 1b, it was confirmed that nanowires were grown vertically along the GaN (002) direction on the Si (111) substrate by XRD measurement. A TEM image of a high aspect ratio single nanowire structure is shown in Figure 1c. The HR-TEM lattice image of Figure 1d showed that the nanowires had high-crystalline quality, and the spacing between two adjacent fringes was about 0.518 nm corresponding to the <0001> direction which is the c-axis crystal direction of GaN. In addition, the selected area diffraction (SAED) measured from the ⟨2−1−10⟩ zone axis shows the result consistent with the crystal growth direction of the GaN nanowires.

In typical InGaN structures, it is difficult to grow a *long*-InGaN layer because of the inhomogeneous distribution and phase separation of In, and changes in crystal nucleation with increasing the nanowire length. Moreover, the doping of *n*-type or *p*-type is more challenging in these *long*-InGaN structures. However, uniformly doped *long*-InGaN nanowire structures are essential for a large-area emission in photonics and photocatalytic reaction in solar water splitting. To overcome this limitation, we introduced the 3-step nanowire epitaxial growth technique capable of *n*-type or *p*-type doping in long-InGaN structure. Since the nanowire structure has a high-volume ratio, it has the advantage of increasing the concentration of In, Mg, and Si by improving the diffusion and nucleation of adatoms.

**Figure 2:**
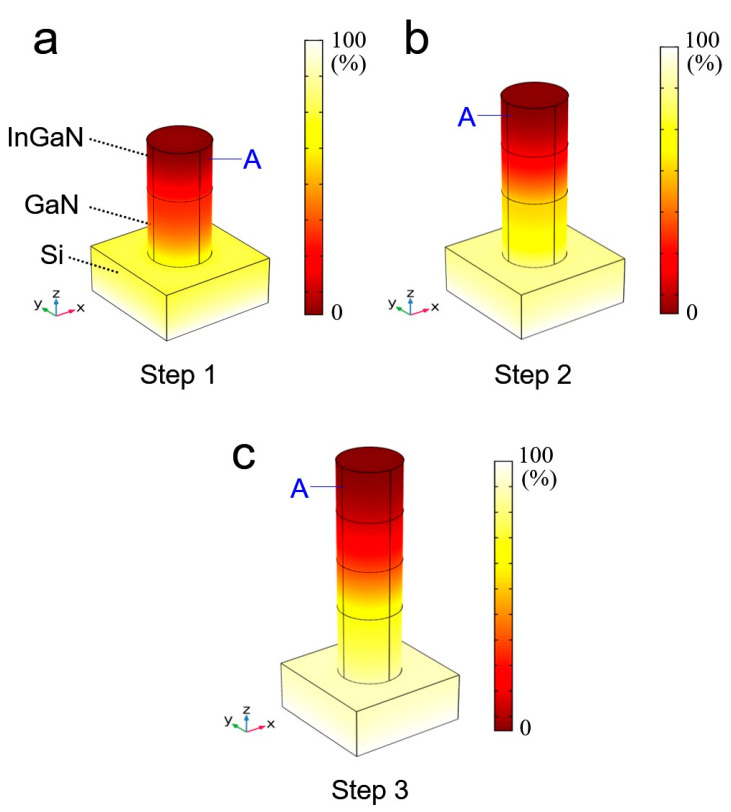
A heat transfer simulation of 3-step modulation growth with *long*-indium gallium nitride (InGaN) on GaN nanowire structures. (**a**) Step 1: 715 °C with 50 nm length, (**b**) Step 2: 719 °C with 100 nm length, (**c**) Step 3: 723 °C with 150 nm length, respectively.

In order to design the *long*-InGaN nanowire heterostructure, we performed a heat transfer simulation using a commercial software package Comsol Multiphysics 5.3a. The InGaN nanowire structure was fabricated on Si substrate by introducing a 3-step modulation temperature growth technique, shown in Figure 2. The diameter and height of a single nanowire were set at 50 nm and 220 nm, respectively. The thermal conductivity of InGaN is assumed to be 10 W/(m·K). The heat transfer simulation was performed by modulating the substrate temperature, and the substrate temperatures and nanowire lengths were set as Step 1: 715 °C with 50 nm, Step 2: 719 °C with 100 nm, Step 3: 723 °C with 150 nm, respectively. All heat flow was transferred from the bottom of the Si substrate to the top of the nanowire. As shown in the simulation results, the GaN part shows a non-uniform temperature distribution in Step 1. On the contrary, a relatively uniform temperature distribution is shown in the InGaN region. This temperature imbalance becomes more severe in Step 2. The temperature distribution varies in the bottom parts except for InGaN on the top, shown in Figure 2b. However, when the substrate temperature is slightly raised, the heat is transferred to the top end of the nanowire, resulting in the same temperature distribution as the surface temperature of Step 1. Such uniform temperature distribution on the nanowire top surface will induce the uniform formation of the InGaN layer by introducing highly stable In incorporation. The modulation temperature of 723 °C in Step 3 can further support the same stable surface temperature as the temperature in Step 1 and Step 2. Figure 2c shows the temperature distribution in the third stage. These simulation results suggest that the new three-step modulated growth technique can be handily used for uniform doping and formation of *long*-InGaN nanowire structures.

**Figure 3:**
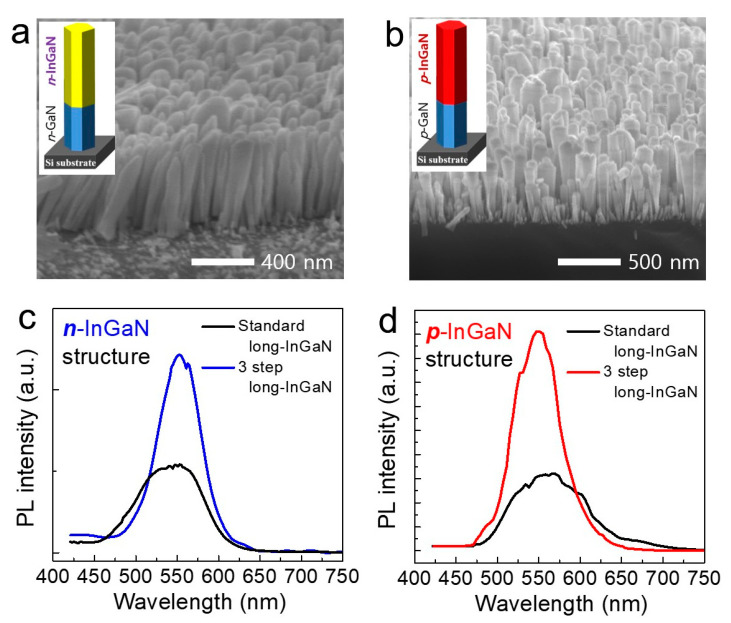
(**a**) A 45°-tilted FE-SEM image of high-quality long *n*-InGaN: Si nanowires grown by 3-step modulation temperature growth (inset: long *n*-InGaN/*n*-GaN structure on Si substrate). (**b**) FE-SEM image of high-quality long *p*-InGaN: Mg nanowires grown by 3-step modulation temperature growth (inset: long *p*-InGaN/*p*-GaN structure). (**c**) Room-temperature photoluminescence spectra of standard long *n*-InGaN:Si nanowire and 3-step long *n*-InGaN:Si nanowire. (**d**) Photoluminescence spectra of standard long *p*-InGaN:Mg nanowire and 3-step long *p*-InGaN:Mg nanowire.

Based on the simulation results, have performed the 3-step epitaxial growth using the MBE system. Firstly, the growth condition includes Ga BEP of ~5.3 × 10^−8^ Torr, substrate temperature of 710 °C, and Si cell temperature of 1170 °C in N_2_ atmosphere of 0.5 sccm for the high-quality *n*-GaN layer. Secondly, in order to distribute the uniform In concentration in the *long*-InGaN nanowire structure, the 3-step epitaxial growth with the modulating temperature was introduced. In Step 1, the substrate temperature was increased from 713 °C to 717 °C for 15 min with an In BEP of 5.5 × 10^−8^ Torr, and a Si cell temperature of 1165 °C for *n*-InGaN nanowire segment. In the second InGaN growth step (Step 2), the In BEP of 6.5 × 10^−8^ Torr and the Si cell temperature of 1170 °C were used with the substrate temperature increased from 717 °C to 721 °C for 15 min. In the last third step (Step 3), the In BEP of 8.0 × 10^−8^ Torr and a Si cell temperature of 1175 °C were used, and the substrate temperature was changed from 721 °C to 723 °C for 15 min. Here, the reason for increasing the In BEP and Si cell temperatures is to minimize the reduction of In incorporation and Si doping at the nanowire top surface. During the epitaxial growth, the nitrogen and plasma power was fixed at 1.2 sccm and 350 W. As shown in Figure 3b, we also grew long *p*-InGaN nanowire structures. All growth conditions were kept the same as above. Here, Mg cell temperatures of 315, 320 and 325 °C were used during the 3-step epitaxial growth.

The FE-SEM images of high-density long *n*-InGaN and long *p*-InGaN nanowires are shown in Figure 3a,b. The nanowires show separate contrasts, which can be expected to be formed of two layers of GaN and InGaN. In order to analyze the optical properties of the long *p*-InGaN nanowires, we investigated the wavelength emission characteristics of the samples using the photoluminescence (PL) system. The PL results indicated that the long *n*-InGaN and long *p*-InGaN nanowires have a wavelength of about 550 nm, shown in Figure 3c,d. It was confirmed that the emission intensities were dramatically improved by ~3 times or more compared to standard short-length InGaN structure. In the standard samples, the substrate temperature and the indium cell temperature were kept constant during the epitaxial growth. Here, we want to compare how much the efficiency of the 3-step temperature modulation technique is improved compared to the nanowire sample grown by the conventional method. This result suggests the suppression of In inhomogeneous distribution was achieved by the new 3-step nanowire growth technique.

**Figure 4:**
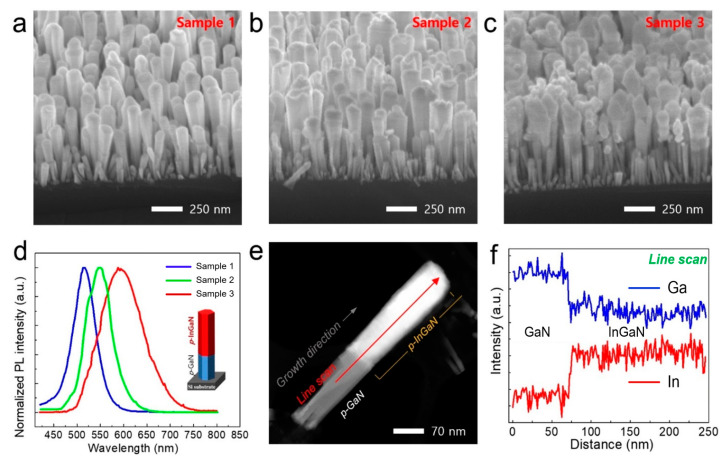
(**a**–**c**) 45°-tilted view FE-SEM images of long *p*-InGaN:Mg nanowires, which were grown at different substrate temperatures (Sample1: 713~723 °C, Sample 2: 693~705 °C and Sample 3: 678~690 °C, respectively) (**d**) Normalized PL spectra of Sample 1, Sample 2 and Sample 3, respectively. (**e**) STEM-HAADF image of a representative long *p*-InGaN:Mg nanowire. (**f**) Energy-dispersive x-ray spectroscopy (EDS) line profile of the long *p*-InGaN:Mg nanowire scanned along the red line.

Long-wavelength emitting *p*-InGaN nanowires are very important but still challenging. So as to increase the concentration of In in the *p*-InGaN nanowire structure, we reduced the temperature range of the substrate to Sample 2: 693~705 °C and Sample 3: 678~690 °C under the same growth condition of Sample 1. Figure 4a–c show the FE-SEM images of the nanowires grown from different substrate temperatures. By increasing the In concentration, the morphology of the nanowires changes very roughly, and the coalescence occurred on the top of the nanowires. It is expected that the lateral growth effect was occurred due to the low substrate temperature, and the defect density was also increased owing to the high In concentration. Figure 4d shows the PL results of three samples. The *p*-InGaN nanowires of Sample 1 have a wavelength of about 514 nm. Sample 2 and Sample 3 were red-shifted with wavelengths of 550 nm and 595 nm, respectively. These results suggest that the 3-step modulation temperature growth method is useful to fabricate *long*-InGaN and to increase In incorporation in the nanowire structure. Figure 4e,f represent a STEM image and an EDS line profile measured from a single long *p*-InGaN nanowire, to confirm the inhomogeneous distribution of In. Figure 4e shows a single *p*-InGaN nanowire image measured by STEM. Along the growth direction of the c-axis, the dark part is *p*-GaN and the bright part is *p*-InGaN, showing a distinct difference in atomic number contrast. The EDS line profile confirms that a highly uniform In concentration is distributed over the entire InGaN nanowire structure, shown in Figure 4f. The newly developed 3-step modulation growth technique is expected to be highly useful for fabricating a uniform long InGaN structure.

**Figure 5:**
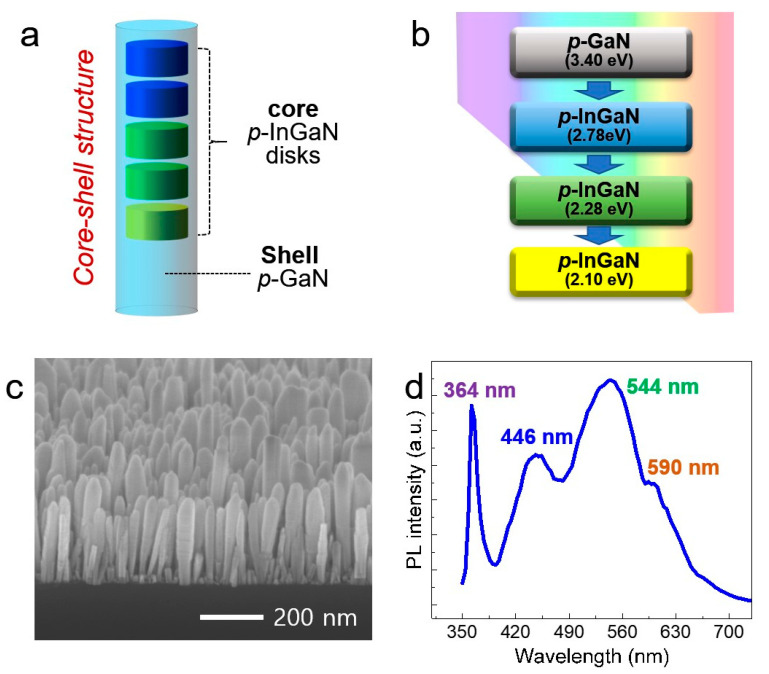
(**a**) Schematic of multi-band *p*-InGaN/*p*-GaN core–shell nanowire heterostructure. (**b**) Schematic of the multi-band InGaN heterostructure for emitting the UV, blue, green, and red spectral range. (**c**) A 45° tilted FE-SEM image of multi-band *p*-InGaN/*p*-GaN heterostructures grown on *p*-GaN nanowires on Si substrate. (**d**) Photoluminescence spectra of the multi-band InGaN/GaN nanowire arrays measured at room temperature.

It is also important that the fabrication of InGaN nanowire heterostructures with various bandgaps that emit/absorb the entire visible wavelength region. We designed *p*-InGaN nanowire heterostructures which have a core–shell structure with the multi-band structure, shown in Figure 5a. It is expected that four multi-band nanowire structure can emit a full wavelength range from UV to visible, as shown in Figure 5b. Firstly, the *p*-InGaN nanowire segment was grown at 685 °C to enhance light emission in the orange and red wavelength ranges. Secondly, the green band wavelength was covered by the substrate temperature of 700 °C. In the last step, *p*-InGaN nanowires that have a blue-light emission were grown at 720 °C. Figure 5c shows the FE-SEM image of core–shell *p*-InGaN/*p*-GaN nanowire heterostructures with the multi-band wavelengths. The uniform nanowires were formed with a high-aspect-ratio. The PL spectrum measured at room temperature verified that the multi-band structure consisting of *p*-GaN and ternary-band InGaN segments has optical emission wavelengths of 364 nm, 446 nm, 544 nm, and 590 nm, respectively, shown in Figure 5d. Such nano-structure will be capable of harvesting or emitting all sunlight in the UV, blue, green, and red regions from the single nanowire heterostructure.

**Figure 6:**
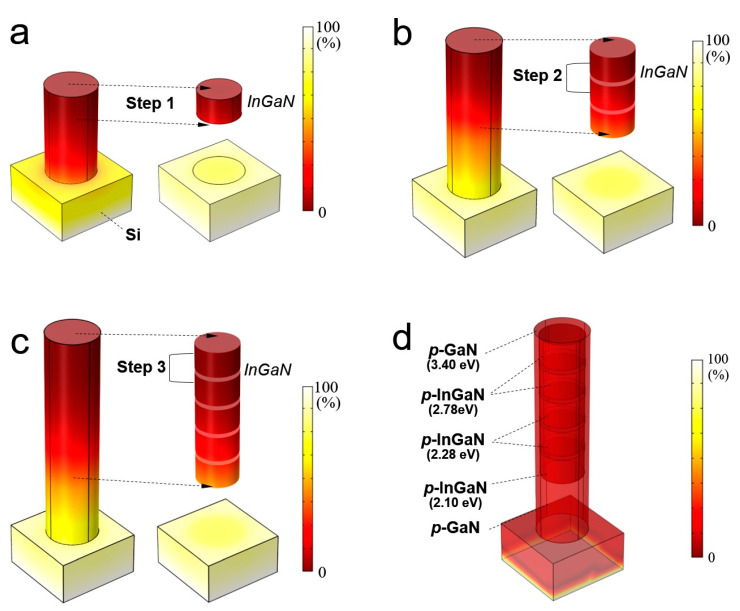
A heat transfer simulation of 3-step modulation growth with multi-band heterojunction nanowire structures. (**a**) Step 1: 685 °C for first InGaN segment, (**b**) Step 2: 700 °C for second InGaN segments, and (**c**) Step 3: 720 °C for third InGaN segments (left: unhidden core–shell structure and right: hidden core-shell structure), respectively. (**d**) Transparent image of multi-band heterojunction nanowire structure which has a wide range of bandgap from 2.10 eV to 3.40 eV.

In order to understand the mechanism for the formation of InGaN segments in multi-band heterojunction nanowire structures, we performed thermal simulation analysis on InGaN structures using the commercial software package Comsol Multiphysics 5.3a. As shown in Figure 6, the nanowire fabrication was also performed by the three-step epitaxial growth technique. The heat was injected through the Si substrate, and the thickness of each layer was set to about 30 nm. The thermal conductivity of GaN is ~130 [W/(m·K)] and the thermal conductivity of InGaN was ~10 [W/(m·K)]. The temperatures for each step were set to Step 1: 685 °C, Step 2: 700 °C, and Step 3: 720 °C, respectively. The simulation results show that each segment has the same temperature distribution on the top surface of the nanowire. Especially, the last Step 3 exhibits nearly uniform temperature distribution on the last two layers of InGaN. These results verify that the temperature modulation technique can readily fabricate the highly uniform multilayer structure. The simulation analysis also shows that high-quality multi-band can be realized in *long*-InGaN nanowire structures by such modulation technique.

**Figure 7:**
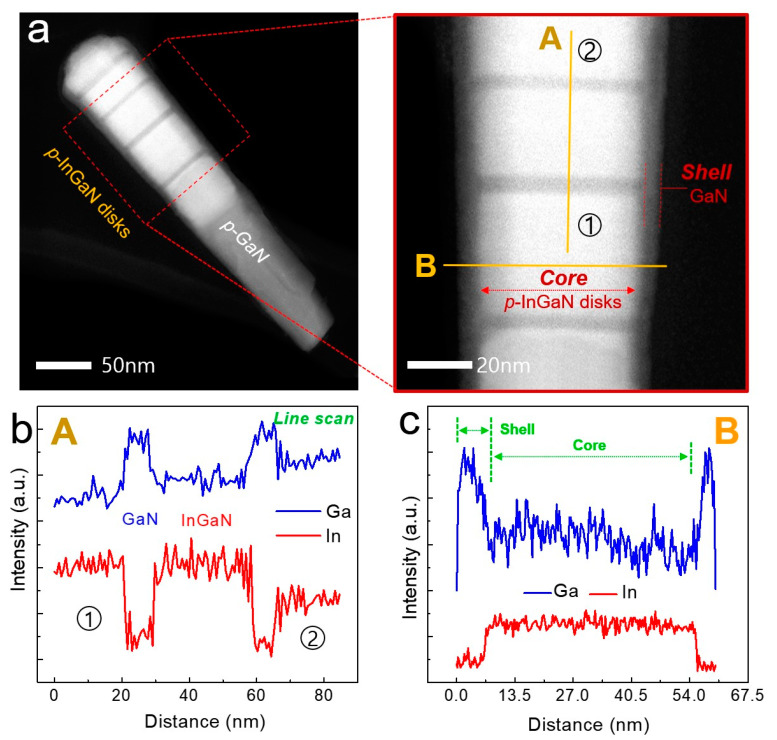
(**a**) STEM-HAADF image of multi-band *p*-InGaN/GaN core–shell heterostructure nanowire. the right image: high-magnification STEM image of the core *p*-InGaN/shell GaN region. (**b**) EDX line profile of the multi-band *p*-InGaN/GaN disks measured along the line labeled with “A”. (**c**) EDX line profile of the core-shell *p*-InGaN/GaN structure measured along the line labeled with “B”.

It is necessary to protect the InGaN active region from surface recombination that interferes with carrier transport, to improve the emission quantum efficiency. In order to protect the InGaN region, we introduced the InGaN/GaN core–shell structure in the nanowire system. Adatom incorporations in the nanowire epitaxy consist of directly impingent adatoms as well as adatoms migrated from the lateral surfaces of the nanowire. Since Ga adatoms have a relatively longer diffusion length than In adatoms at the surface, the Ga adatoms can easily reach the top surface of the nanowires, whereas the In adatoms rarely reach, and surface desorption easily occurs on the top lateral surface. As a result, a very thin GaN layer can be formed on the lateral surface of the nanowire. Such GaN thin layer surrounding the InGaN core segment will protect the InGaN active region leading to emission efficiency improvement. The structural characteristics of the core–shell *p*-InGaN/*p*-GaN nanowires were analyzed by TEM measurement. The STEM image of the single nanowire demonstrates the five *p*-InGaN/*p*-GaN multi-structures, clearly separated *p*-GaN segments by the atomic number contrast, shown in Figure 7a. The high-magnification STEM image, in Figure 7b, shows the more clearly separated *p*-InGaN structure which is a core–shell structure surrounded by *p*-GaN. We also measured the EDX line profile on “A” and “B” markers to confirm the structure formation of core-InGaN and shell-GaN. As shown in Figure 7c, the EDS line profile proves that the In and Ga components are clearly separated and formed along the <0001> growth direction. In addition, the *p*-InGaN segments showed a high In concentration in the “1” part and a low In concentration in the “2” part, which is consistent with the PL result of Figure 5d. Moreover, it was confirmed that the GaN shell structure was only formed on the nanowire surface. The EDS line profile in part “B” shows the high In concentration only in the core, and the Ga signal was detected with high intensity on the sidewall of the nanowire, shown in Figure 7c. The above experimental results verified that we successfully fabricated the high aspect ratio nanowire structure, and that the wavelength of InGaN can be easily adjusted from UV to visible wavelength. Furthermore, it was also confirmed that the core–shell structure can protect the InGaN active region, thereby increasing the emission quantum efficiency. The developed high-quality, high-aspect-ratio InGaN/GaN nanowire heterostructures can be profitably used as the applications of LEDs, LDs, solar cells, and photocatalysts [20,21,22,23].

## 3. Conclusions

We successfully developed high-quality doped InGaN nanowire heterostructures by using the exceptional technique of the MBE system. By introducing the heat transfer simulation modeling, we found the uniform thermal distribution in the *long*-nanowire heterostructures. Compared to conventional nanowire structures, the developed long *n*-/*p*-InGaN nanowires show improved emission efficiency with a high Si/Mg doping effect and high aspect ratio density. The 3-step modulation growth method was introduced to synthesize a highly uniform In concentration. The results showed that the emission wavelength of *p*-InGaN nanowires can be varied from 514 nm to 595 nm by changing the substrate temperature. FE-SEM images verified that the nanowires were vertically grown on the Si substrate with various shapes. Moreover, we also demonstrated the high-quality multi-band *p*-InGaN/GaN heterostructure can be fabricated by the 3-step modulation growth technique. Such core–shell structure can protect the InGaN active region from the surface recombination. The development of high-aspect-ratio *p*-InGaN nanowires, which have the maximum surface area, is expected to contribute significantly to improved high-efficiency luminescence.
